# Serum levels of irisin and nesfatin-1 in multiple sclerosis

**DOI:** 10.1590/0004-282X-ANP-2020-0520

**Published:** 2022-02-06

**Authors:** Mustafa ALTAŞ, Ali Ulvi UCA, Turan AKDAĞ, Faruk Ömer ODABAŞ, Osman Serhat TOKGÖZ

**Affiliations:** 1 Necmettin Erbakan University, Meram Faculty of Medicine, Department of Neurology, Konya, Turkey. Necmettin Erbakan University Meram Faculty of Medicine Department of Neurology Konya Turkey; 2 Necmettin Erbakan University, Meram Vocational School, Konya, Turkey. Necmettin Erbakan University Meram Vocational School Konya Turkey; 3 University of Health Sciences, Konya City Hospital, Department of Neurology, Konya, Turkey. University of Health Sciences Konya City Hospital Department of Neurology Konya Turkey

**Keywords:** Multiple Sclerosis, Irisin, Nesfatin-1, Inflammation, Apoptosis, Oxidative Stress, Esclerose Múltipla, Irisina, Nesfatina-1, Inflamação, Apoptose, Estresse Oxidativo

## Abstract

**Background:**

Multiple sclerosis (MS) is an inflammatory and neurodegenerative autoimmune chronic neurological disease. Currently, there are no effective serum biomarkers to verify MS diagnosis, to assess disease prognosis, and evaluate response to MS treatment.

**Objective:**

The present study is a preliminary assessment of irisin and nesfatin-1 serum levels in patients with relapsing-remitting MS (RRMS).

**Methods:**

A total of 86 participants, 42 patients with RRMS diagnosis and 44 healthy controls were included in the study. The serum irisin and nesfatin-1 parameters of the patients and control group members were analyzed.

**Results:**

Irisin and nesfatin-1 levels of the RRMS patients were significantly lower than the controls (z: -3.82, p<0.001; z: -4.79, p<0.001, respectively) The cut-off level of irisin is 10.390 (ng/mL) (sensitivity: 84.1%, specificity: 71.4%, AUC: 0.800), and the cut-off level of nestatin-1 is 7.155 (ng/mL) (sensitivity: 68.2%, specificity: 64.3%, AUC: 0.739) in the ROC analysis. For these cut-off levels in the case-control groups, the lower irisin and nesfatin-1 levels are the independent variables for MS patients (OR 9.723, 95%CI 2.884-32.785, p<0.001; OR 3.992, 95%CI 1.336-11.928, p<0.001) respectively.

**Conclusion:**

The present study revealed lower irisin and nesfatin-1 levels in patients with RRMS. These findings suggest that the decreased levels of irisin and nesfatin-1 peptides may contribute to MS pathogenesis such as inflammation, oxidative stress, and apoptosis in MS, leading to demyelination, axonal damage with neuronal loss, and gliosis.

## RESUMO

**Antecedentes:**

A esclerose múltipla (EM) é uma doença neurológica crônica autoimune inflamatória e neurodegenerativa. Atualmente, não há biomarcadores séricos eficazes para verificar o diagnóstico de EM, para avaliar o prognóstico da doença e avaliar a resposta ao tratamento de EM.

**Objetivo:**

O presente estudo é uma avaliação preliminar dos níveis séricos de irisina e nesfatina-1 em pacientes com EM recorrente-remitente (EMRR).

**Métodos:**

Um total de 86 participantes, 42 pacientes com diagnóstico de EMRR e 44 controles saudáveis, foram incluídos no estudo. Os parâmetros séricos de irisina e nesfatina-1 dos pacientes e membros do grupo controle foram analisados.

**Resultados:**

Os níveis de irisina e nesfatina-1 dos pacientes com EMRR foram significativamente mais baixos do que os dos controles (z: -3,82, p <0,001; z: -4,79, p <0,001, respectivamente). O nível de corte de irisina é 10,390 (ng/mL) (sensibilidade: 84,1%, especificidade: 71,4%, AUC: 0,800), e o nível de corte de nestatina-1 é 7,155 (ng/mL) (sensibilidade: 68,2%, especificidade: 64,3%, AUC: 0,739) na análise ROC. Para esses níveis de corte nos grupos de caso-controle, os níveis mais baixos de irisina e nesfatina-1 são as variáveis independentes para pacientes com EM (OR 9,723, IC95% 2,884-32,785, p <0,001; OR 3,992, IC95% 1,336-11,928, p <0,001) respectivamente.

**Conclusão:**

O presente estudo revelou níveis mais baixos de irisina e nesfatina-1 em pacientes com EMRR. Esses achados sugerem que os níveis diminuídos de peptídeos irisina e nesfatina-1 podem contribuir para a patogênese da EM, como inflamação, estresse oxidativo e apoptose na EM, levando à desmielinização, dano axonal com perda neuronal e gliose.

## INTRODUCTION

Multiple sclerosis (MS) is a chronic disease defined by its neurodegenerative and autoimmune inflammatory character with multifocal inflammation sites due to autoreactive T and B-lymphocytes and macrophage infiltrations leading to demyelination, axonal damage with neuronal loss, and gliosis in both the white and the gray matter of the central nervous system (CNS)^1^.

Biomarkers for the prognosis and definitive diagnosis of MS have until recently been limited with cerebrospinal fluid analyses. To our best knowledge, there are no effective serum biomarkers for the definitive diagnosis of MS, prognosis assessment, and evaluation of the patients’ responses to the treatment^2^. Currently, periodic scanning of MS patients by Magnetic Resonance Imaging (MRI) is an important tool for monitoring disease activity and response to treatment^3^. However, in evaluating disease progression and determination of prognosis, this expensive, time-consuming, and semi-quantitative imaging marker has limited sensitivity^4^.

Irisin has been determined as a skeletal muscle-originated myokine with increasing serum levels during exercise in order to provide the energy necessary and glucose homeostasis through the stimulation of white adipose tissue browning^5^. But new investigations have revealed that irisin acts as both an adipokine^6^ and as a potential neurokine^7^. It is well documented that irisin plays a significant role in apoptosis, inflammatory, and oxidative stress^8^. In a study performed by Bosma et al.^9^ in mice FNDC4 (homology with FNDC5) may have an anti-inflammatory influence on macrophages and thus could improve colitis. New studies have noted increased or decreased irisin levels in various diseases. A study conducted by Ebert et al.^10^ reported that serum irisin level was lower in chronic kidney disease. Choi et al.^11^ also reported that it was lower in patients with type 2 diabetes mellitus (DM). However, Ates et al.^12^ demonstrated that irisin level was higher in patients with type 1 DM. Current studies have revealed reduced serum irisin levels in breast cancer cases^13^, with inhibitory effect on malignant breast cancer cells^14^. However, the physiological properties and functional roles of irisin in the brain have yet to be fully explained. Irisin is determined to be involved in metabolism regulation, neuronal differentiation, and energy expenditure besides ischemia-induced neuronal injury preserving function^15^. Moreover, a recent study has shown that a low level of serum irisin was a possible biomarker in the early prediction of ischemic stroke^16^. Recent data suggest that irisin is expressed in the brain and induces brain-derived neurotrophic factor (BDNF) expression in rat hippocampus, thus increasing brain irisin levels could preserves memory and hinders cognitive destruction in an Alzheimer’s disease rat model study^17^.

Nesfatin-1, a potent anorexigenic peptide playing an important role in the regulation of feeding homeostasis and energy expenditure, was first defined in 2006^18^^,^^19^. It is obtained from the precursor-peptide NEFA/nucleobindin 2 (NUCB2), present not only in the CNS but also in the periphery and reaching the brain via non-saturable transmembrane diffusion^20^. Posttranslational modification of NUCB2 by prohormone convertase produces three cleavage products, i.e. nesfatin-1, nesfatin-2, and nesfatin-3^19^. Nesfatin-1 intracerebroventricular injection or intraperitoneal application to mice resulted with reduced food intake^18^^,^^21^. The recently discovered fact that nesfatin-1 expresses neuron activation in the brainstem and hypothalamus highlights the potential role of these neurons in the transfer of information received from circulating immune factors^22^. Furthermore, nesfatin-1 has also been tested as a therapeutic agent in the treatment of infectious and autoimmune diseases. There are also some reports about nesfatin-1 effects in psychiatric disorders and neurogenic diseases. In patients with major depressive disorder^23^ or epilepsy^24^ elevated nesfatin-1 levels and in patients with generalized anxiety disorder^25^ depleted nesfatin-1 levels were determined. Interestingly, another study reported elevated nesfatin-1 levels in schizophrenia patients^26^. Current studies revealed that in traumatic rat brains nesfatin-1 has anti-inflammatory and antiapoptotic effects^27^. Nesfatin-1 has also been observed to have a significant effect on the suppression of brain damage resulting from oxidative mechanisms^28^^,^^29^.

To our best knowledge, there are no studies investigating irisin and nesfatin-1 levels in MS patients. The aim of the present study is to contribute to the existing literature by assessing serum irisin and nesfatin-1 levels, both known to have similar anti-inflammatory, antioxidant and antiapoptotic effects and to determine whether serum irisin and nesfatin-1 levels may be used as a biomarker for making MS diagnosis, and evaluating course of disease and responses to relapsing-remitting MS (RRMS) treatment.

## METHODS

After the approval of the Meram Medical Faculty, Necmettin Erbakan University institutional ethics committee enumerated as 2020/2330 and dated 21^st^ of February 2020, a written consent form was filled out by all participants who were informed in depth about the study to be conducted. The present study is conducted according to the Helsinki good clinical practice guidelines.

### Participants

Between March 2020 and September 2020, MS patients admitted to the Necmettin Erbakan University, Faculty of Medicine, Neurology MS Outpatient Clinic for control purposes were considered for the present study. Out of the MS patient population, 42 consecutive RRMS patients were selected according to the inclusion criteria. The control group consisted of 44-age and gender matched-individuals without any previous health conditions. Forty patients (95.2%) in the RRMS group were receiving disease modifying drugs including daily oral teriflunomide 14 mg intake (5 patients), subcutaneous interferon beta-1b every other day (5 patients), subcutaneous interferon beta-1a 44 mcg three times per week (10 patients), subcutaneous glatiramer acetate 40 mg three times per week (6 patients), daily oral fingolimod 0.5 mg intake (14 patients), and 2 patients (4.8%) were naïve for treatment.

Inclusion criteria for the RRMS patient group were as follows: voluntarily enrolment, age between 18 and 55 years, RRMS diagnosis as described in the 2010 revision to the McDonald Criteria for dissemination of time as well as space and currently without relapse, no reported treatment with pulsed intravenous methylprednisolone within the last 3 months, below 5.5 according Expanded Disability Status Scale (EDSS), lack of any chronic or acute medical condition other than MS as confirmed in former medical reports and clinic examinations; no use of medications including antiaggregants, anticoagulants, corticosteroids, selective serotonin re-uptake inhibitors, antipsychotics, and no report of illicit drug or substance use or addiction.

Gender, age, and clinical data of the patients as well as height and weight of the participants were recorded at the same time. Body mass index (BMI) was calculated as (w/kg)/(h/m^2^).

### Measurement of irisin and nesfatin-1

Blood specimens collected from each participant were centrifuged at 3000xrpm for 15 minutes within 30 minutes subsequent to blood drawal and obtained sera was kept at -80°C until assay. Irisin and nesfatin-1 serum levels were determined through enzyme linked immunosorbent assay (ELISA) technique. The serum concentrations of irisin were analyzed via Human Irisin ELISA kits (Bioassay Technology Laboratory, Shanghai, China, catalog no: E3253Hu). The sensitivity was determined as 0.095 ng/mL; standard curve range as 0,2-60 ng/mL, intra-assay as <8%, and inter-assay as <10%. Nesfatin-1 serum concentrations were analyzed using Human Nesfatin-1 ELISA kits (Bioassay Technology Laboratory, Shanghai, China, catalog no: E3063Hu). The sensitivity was determined as 0.15 ng/mL; standard curve range as 0.3-90 ng/mL, intra-assay as <8%, and inter-assay as <10%. For the assay, manufacturer’s instructions were followed throughout the study. The absorbance of the specimen was measured at 450 nm by absorbance microtiter plate reader with a double-blind procedure (ELx800TM, BIO-TEK instruments, USA).

### Statistical analysis

Data analysis was conducted using *Statistical Package for the Social Sciences* software (version 15.0; SPSS Inc, Chicago, IL). Mean values are presented with standard deviation (±) or median given with range. Kolmogorov Smirnov test was used to test normality. Student’s *t*-tests for parametric comparisons between the patient and control groups, Mann-Whitney U test for nonparametric comparisons, and chi-square test for the comparison of categorical data. Kruskal-Wallis nonparametric test was used for more than two groups.

Receiver operating characteristic (ROC) analysis was used to define areas, Areas Under the Curve (AUC), sensitivity, and specificity, positive and negative predictive values. Binary Logistic regression analysis was conducted to determine independent predictive risk factors for MS. P<0.05 threshold level was taken to determine statistical significance.

## RESULTS

**Figure 1.**
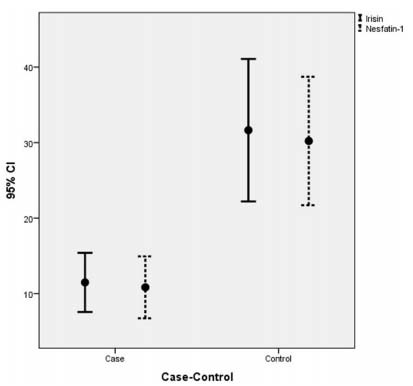
The mean serum irisin and nesfatin-1 levels in the case and control groups.

86 volunteers with a mean age of 38.0±8.9 were included in the study. Table 1 present the demographic and clinical characteristics of the patients and controls. Serum irisin and nesfatin-1 levels were significantly lower in MS patients (Z score: -3.82, p<0.001; Z score: -4.79, p<0.001, respectively) (Figure 1). BMI is significantly higher in the patients group than the controls (z: -5,287, p<0.001).

**Table 1.** The demographic and clinical characteristics of the patients and controls.

	Patients n=42	Controls n=44
Age (year) (mean/±SD)	38.12 (±8.96)	37.3 (±8.9)
Female gender (n/%)	27 (64.3%)	25 (56.8%)
BMI (kg/m^2^) (median/range)	25.8 (19.11)	21.59 (10.46)
Irisin (ng/mL) (median/range)	8.21 (64.45)	14.98 (97.45)
Nesfatin-1 (ng/mL) (median/range)	5.90 (48.01)	19.65 (88.29)
EDSS (median/range)	1.5 (5.50)	
MS duration (year) (median/range)	7.0 (25.0)	
MS relapse number* (median/range)	4.0 (9.0)	

SD: standard deviation; BMI: body mass index; EDSS: Expanded Disability Status Scale; MS: multiple sclerosis. *Number of all relapses since diagnosis.

Serum irisin and nesfatin-1 levels were significantly lower in MS patients (p<0.001).

**Figure 2.**
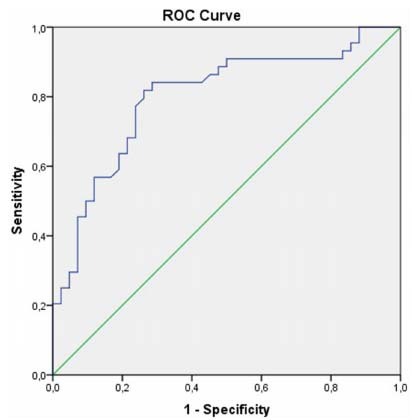
Receiving operating curve of irisin for the prediction of case-control.

**Figure 3.**
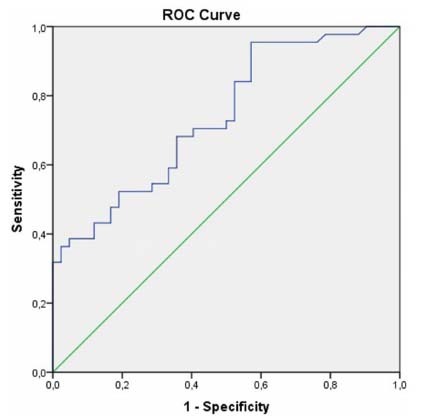
Receiving operating curve of nesfatin-1 to prediction of case-control.

In ROC analysis of irisin (Figure 2) and nesfatin-1 (Figure 3), cut-off levels for MS were 10.390 (ng/mL) (sensitivity: 84.1%, specificity: 71.4%, PPV: 81.08%, NPV: 75.51% AUC: 0.800 (0.704-0.896), and 7.155 (ng/mL) (sensitivity: 68.2%, specificity: 64.3%, PPV: 65.85%, NPV 66.67%: AUC: 0.739 (0.636-0.842) respectively.

Cut-off level: 10.390 (ng/mL) sensitivity: 84.1%, specificity: 71.4%, AUC: 0.800.AUC: areas under the curve.

Cut-off level: 7.155 (ng/mL) sensitivity: 68.2%, specificity: 64.3%, AUC: 0.739. AUC: areas under the curve.

In the regression model, the *Odds Ratio* (OR) was 9.273 (95% confidence interval [95%CI]): 2.884-32.785, p<0.001) when the irisin <10.390 ng/mL is independent of other variables for the RRMS patients. Overall corrected percentage is 81.4% (Table 2). The OR was 3.992 (95%CI 1.336-11.928, p: 0,013) when the nesfatin-1 <7.155 ng/mL is independent of other variables for the RRMS patients. Overall corrected percentage is 80.2% (Table 2).

**Table 2.** Binary logistic regression analysis for Irisin-Nesfatin-1 (case-control groups).

	Irisin			Nesfatin-1		
	p-value	OR	95%CI	p-value	OR	95%CI
Age	0.156	1.052	0.981-1.129	0.177	1.047	0.979-1.120
Gender	0.887	0.916	0.276-3.041	0.751	0.835	0.275-2.540
BMI	0.000	0.643	0.508-0.814	0.000	0.626	0.502-0.779
Irisin <10.390 (ng/mL)	<0.001	9.723	2.884-32.785			
Nesfatin-1 <7.155 (ng/mL)				0.013	3.992	1.336-11.928

Irisin Nagelkerke R^2^: 57.0%; Nesfatin Nagelkerke R^2^: 48.7%; BMI: body mass index; OR: *Odds Ratio*; 95%CI: 95% confidence interval.

No statistically significant correlation could be determined between serum irisin, nesfatin-1 and overall relapses number, age, BMI, EDSS, and disease duration. The comparison of the gender variable and mean irisin and nesfatin-1 serum levels revealed no statistically significant difference (p>0.05). Likewise, there isn’t any significant difference between disease modifying drugs regarding to irisin and nesfatin-1 (p>0.05).

## DISCUSSION

The present study is the first to investigate the association between MS and serum irisin and nesfatin-1 levels. Serum irisin and nesfatin-1 levels were determined in the present study to be significantly lower in the RRMS group than the control group. However, there were no statistically significant correlations between serum irisin and nesfatin-1 levels with age, disease duration, EDSS score, and overall relapse number. This could be an indication that molecular difference in patient group begins probably in the early stages of the disease. In MS, inflammation is the primary cause and recent studies have confirmed the anti-inflammatory effects of irisin and nesfatin-1 on the damaged brain^27^^,^^28^^,^^30^. Considering the inflammatory nature of MS, lower irisin and nesfatin-1 serum levels presence in RRMS patients was surprising. Hence, it might be concluded that decreased irisin and nesfatin-1 expressions may play a role in MS pathogenesis. However; more comprehensive studies are needed to determine the underlying causes of reduced serum irisin and nesfatin-1 levels in MS patients.

The association between irisin-nesfatin-1 levels and several other diseases has been established previously in the relevant literature. Serum irisin concentration is regulated by several factors such as obesity, exercise, diet, pharmacological, and some pathological conditions^31^. In obese people high plasma irisin levels have been determined and the underlying cause was defined to be an adaptive response that attempts to compensate the imbalance in glucose and lipid homeostasis^32^. Similarly, in a study conducted with Chinese DM Type 1 and Type 2 patients and healthy controls, there were nonsignificant statistically positive correlations between plasma nesfatin-1 levels and BMI^33^.

In the present study, both serum irisin and serum nesfatin-1 levels were determined as predictive factors independent of BMI for RRMS disease (Table 2). Whereas serum irisin and nesfatin-1 levels are expected to be higher in MS patients who are heavier than the control group, the low levels of these biomarkers support the relationship between MS and irisin and nesfatin-1. Higher serum irisin levels were found in healthy elderly patients compared to young healthy controls^34^. Irisin is negatively correlated with age since it is a muscle growth promoter^35^. However, Li et al. could not determine a correlation between nesfatin-1 levels and age^33^. No age-related increase in serum irisin and nesfatin-1 levels was observed in the present study. The reason for this outcome could be the presence of young MS patients with a narrow age range in the present study. Circulating irisin levels in younger individuals are higher in females than males^36^. Likewise, higher nesfatin-1 plasma levels were found in women compared to men^37^. However, in the present study, no statistically significant gender related difference could be determined in terms of irisin and nesfatin-1 levels.

In a study conducted with healthy participants by Ruan et al.^38^, the level of circulating irisin was approximately 12.7 times higher than the CSF irisin level. Similarly, in the study of Tan et al.^39^ reported that plasma nesfatin-1 levels were about 3 times higher than the CSF nesfatin-1 levels. In the present study, CSF analysis was not performed, and serum irisin levels of RRMS patients were 1.82 times and serum nesfatin-1 levels 3.33 times lower than healthy controls. Although, detected low serum irisin and nesfatin-1 levels in MS patients give rise to thought that CSF levels can be low also, further studies are needed to determine CSF levels of these peptides in MS patients. However, how the adipo-myokine signal enters the brain, whether the CSF irisin and nesfatin-1 uptake involve a saturable transport mechanism, and the level of central irisin and nesfatin-1 expression in response to diseases still remain mainly unclear^38^^,^^39^. The literature review shows that, circulating irisin and nesfatin-1 levels in healthy individuals and patients indicate significant changes in different series^38^^,^^40^. This heterogeneity can be attributed to the use of different ELISA kits, racial differences, age, gender, BMI, etc.^33^^,^^41^.

The specific elements that provoke MS pathogenesis remain unknown. Evidence suggests that inflammation^27^^,^^28^^,^^30^, apoptosis^42^^,^^43^ and oxidative stress^44^^,^^45^, are important contributors to etiology, progression, and clinical symptoms of MS. In this regard, the literature above implies that the decreased irisin and nesfatin-1 concentrations could have a significant role in MS development. In the present study, in RRMS patients a higher percentage of lower irisin and nesfatin-1 levels have been observed compared to the healthy controls below the irisin 10.390 (ng/mL) threshold levels and nesfatin-1 7.155 (ng/mL) threshold level (9.72; 3.99 times respectively). This finding could help to improve understanding of MS and enhance strategies of treatment.

However, there are some inherent limitations of the present study. First of all, the study population is relatively small. Second, only patients without relapse were included in the RRMS group. Therefore, the results of the present study should be verified with studies conducted on progressive MS patients and during relapse in RRMS. In the present study, only irisin and nesfatin-1 serum levels were measured in MS patients and the control group. Determining changes in CSF irisin and nesfatin-1 levels will greatly contribute to understanding MS pathogenesis. Moreover, further studies with increased patient series, with a prolonged evaluation process, and assessment of post-treatment levels could enable a comprehensive conception of the cause-effect relationship of irisin and nesfatin-1 peptides in MS.

In conclusion, the present study revealed lower irisin and nesfatin-1 levels in patients with RRMS. These findings suggest that the decreased levels of irisin and nesfatin-1 peptides may contribute to MS pathogenesis such as inflammation, oxidative stress, and apoptosis in MS, leading to demyelination, axonal damage with neuronal loss, and gliosis. The present study will most probably pave the way for further studies on serum irisin and nesfatin-1 levels to investigate their potential use as treatment options and the possibility to prevent or even slow down RRMS progression.
